# Repurposed Medicines: A Scan of the Non‐commercial Clinical Research Landscape

**DOI:** 10.1002/prp2.70049

**Published:** 2024-12-16

**Authors:** Sola Akinbolade, Ross Fairbairn, Alex Inskip, Rhiannon Potter, Aoife Oliver, Dawn Craig

**Affiliations:** ^1^ National Institute for Health and Care Research (NIHR) Innovation Observatory, Population Health Sciences Institute, Faculty of Medical Sciences Newcastle University Newcastle UK

**Keywords:** academies and institutes, clinical trial, drug repositioning, drug therapy, healthcare research, therapeutic use

## Abstract

Medicine repurposing is a strategy to identify new uses for the existing medicines for the purpose of addressing areas of unmet medical need. This paper aims to provide horizon scanning intelligence on repurposed medicines that are evaluated by non‐commercial organizations such as academia and highlights opportunities for further research to improve patient health outcomes. A scan of the clinical landscape of non‐commercially sponsored repurposed medicines is routinely conducted by the NIHR Innovation Observatory (IO). This ongoing project involves a horizon scan of clinical trial registries and the IO's internal horizon scanning Medicines Innovation Database to identify potential candidate medicines used as monotherapy or in combination to treat new indications outside the scope of their licensed indication. In addition to making these data publicly available, the output also supports the NHS England Medicines Repurposing Programme. The snapshot scan reported here (trials completing April 2020–March 2023) identified a total of 528 technologies (meaning, a single product or combination of medicinal products targeting a specific indication in one or more related trials). The technologies were classified according to their characteristics and targeted therapeutic indications as well as revealing the least treated disease conditions. The candidate medicines identified in this scan could potentially receive tailored support toward adoption into practice and policy. The NIHR IO regularly provides this scan as a source of intelligence on repurposed medicines. This provides valuable insights into innovation trends, gaps, and areas of unmet clinical need.

## Graphical abstract

Top ten therapeutic areas targeted in the repurposed medicines scan.

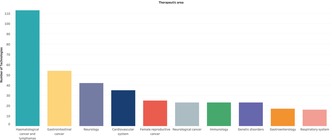

## 1 Introduction

Drug discovery and development require an adequate understanding of the intended disease condition, identification of potential candidate molecules, established methods for drug production, and the initiation of clinical trials to test the efficacy, effectiveness, and safety of the drug [1]. The journey of new drug discovery and the overarching target to address unmet clinical needs imposes major challenges to the pharmaceutical industry [2, 3]. The potential for serious unacceptable adverse effects or inadequate efficacy profiles, coupled with long timelines and high cost implications, limit the successful development of new active substances [4, 5]. If substantial reductions in the process, associated costs, and time for drug development could be realized, this would provide clinicians and patients with early access to effective medicines.

Medicine repurposing, also known as drug repositioning, can be defined as a strategic process of identifying new uses or indications for approved medicines beyond their original indication [3]. The repurposing strategy serves as an opportunity to address areas of unmet treatment need in a timely manner while reducing costs and associated risks of novel drug development [6]. For example, the urgent need for effective treatments for COVID‐19 required the early discovery of suitable medicines that showed great promise to treat the disease, and this led scientists to use repurposing strategies [7]. Many years of research have led to the discovery of the potential for the existing medicines to treat conditions outside their licensed indications [8]. In the 1980s, the analgesic aspirin was repurposed as an antiplatelet aggregation drug for cardiovascular disorders and, more recently, has been investigated for cancer prevention. Sildenafil, an investigative drug for the treatment of coronary artery disease, hypertension, and angina pectoris, produced unexpected side effects, which led to its repurposed indication for the treatment of erectile dysfunction [8, 9].

The concept of medicine repurposing has been pursued not only by the pharmaceutical industry, but also by research institutes and academia [10]. Academic and investigator‐initiated research projects are usually focussed on the achievement of a scientific breakthrough that could improve patients' health and attract funding or partnerships, rather than a commercial gain or the accomplishment of a strategic business model [10]. However, by exploring repurposing without industry collaborations, academic researchers typically face a number of financial, infrastructural, operational, and regulatory hurdles [11, 12]. The lack of access to preclinical and clinical data on discontinued/failed candidate molecules or drugs protected by patents held by pharmaceutical companies restricts the exploration of potential candidate molecules to generic (off‐patent) drugs [10]. Further, the financial burden of conducting investigator‐led studies has been identified as one of the major barriers to repurposing [12]. It is worth noting that, while a pharmaceutical company (as a license holder for an approved medicine) would only need to submit a clinical variation to include a new indication, a non‐commercial organization (i.e., academic researchers) would be required to follow a lengthy full application process to obtain approval or marketing authorization (MA) for a candidate medicine and may not be able to fulfill the legal, financial, and regulatory responsibilities of a license holder [13]. These issues and requirements can be challenging to academic researchers, which in turn may hinder the development of the repurposed medicine or stop the exploration in the first instance.

The establishment of collaborations between pharmaceutical companies and academic researchers appears to offer an opportunity to overcome many of these hurdles [3, 10]. These partnerships could provide a platform to combine expertise in drug research with experience in commercial drug development [10]. The initiation of government‐sponsored schemes could also provide support and promote repurposing opportunities for non‐commercial organizations in terms of providing financial incentives, supporting evidence generation and adoption into the healthcare system [13].

Despite the hurdles outlined, there are many clinical trials being conducted and sponsored by non‐commercial organizations hoping to enable access to new treatment options for the benefit of public health. Given the significant unmet need in some disease areas, there is an increased focus on medicines repurposed to meet these needs; this activity is mostly driven by academia or research institutes [12]. However, there is a growing pool of candidate medicines which are not being pursued for repurposing by pharmaceutical companies either due to failed efficacy in previous studies or irrelevance to their business model [3]. These potential candidate medicines could provide opportunities for academia to explore and discover new indications to which the medicine may be used.

The aim of this paper is to provide horizon scanning data and intelligence on repurposed medicines in clinical development by non‐commercial organizations and offer valuable insights for future medicine repurposing research as well as potential tailored support toward their adoption into practice and policy to improve patient health outcomes.

## 2 Method

In 2022, the National Institute for Health and Care Research (NIHR) Innovation Observatory (IO) began an ongoing project in collaboration with the NHS England Medicines Repurposing Programme (MRP) to identify repurposed medicines in clinical development by non‐commercial organizations [13]. The project involves horizon scanning, a method for identifying emerging healthcare technologies to support proactive decision‐making [14], by analyzing clinical trial registries (ClinicalTrials.gov and EU Clinical Trials Register) to identify potential candidate medicines, either used as monotherapy or in combination, being investigated for new therapeutic indications outside their approved or licensed uses [15].

A detailed search process using trial registries was developed to identify clinical trials of potential repurposed candidates. The following specific inclusion criteria were applied to trial data: trial phase: *phase I/II to III*; primary sponsor: *non‐commercial (any institution other than commercial pharma companies)*; primary completion date: *within a specific range (01/04/2020–31/03/2023 for the initial scan)*; trial location: *at least one site in USA, European Union, United Kingdom, Australia, or Canada*; number of trial sites: *at least two (records with blank locations were considered ambiguous and included)*; condition: *any condition other than COVID‐19 (to ensure other disease areas are not overshadowed)*.

Some of these criteria were applied through the search functionality of the trial registries. The data was then exported into spreadsheet software for additional automated checks before identifying trial records that required manual review. The primary focus of the manual assessment was on the type and status of the interventions. The interventions needed to be primarily pharmaceutical, excluding devices, surgical procedures, or behavioral interventions. Complementary and alternative medicines (CAM), herbal remedies, traditional Chinese medicines (TCM), and dietary supplements were also not included. Crucially, all interventions had to be “repurposed”—meaning they were licensed but being tested for an indication different from that specified in their existing license. This was determined based on the medicine's current UK MA status, verified through the Electronic Medicines Compendium (EMC) [16] or Medicines and Healthcare products Regulatory Agency (MHRA) [17] websites.

The IO maintains an internally facing resource known as the “Medicines Innovation Database” (MInD)—a horizon scanning database populated with data from clinical trial registries, direct engagement with companies, news media, and UK PharmaScan [18]. MInD serves as a resource to inform and support national stakeholders and policymakers in their decision‐making processes. While the inclusion criteria for adding trials to MInD share some similarities with those for the repurposed medicines project, MInD focuses more on trials that could lead to commercialization and new UK licenses. Some trials identified during the search for repurposed medicines were already listed on MInD but were still reassessed for relevancy through a different evaluation process. Other trials required a more thorough screening before being added to MInD for future reference. Unsurprisingly, most trials included in the final scan were not previously on MInD, due to the differing focus between standard MInD inclusion criteria and those of the repurposed medicines project.

Following the completion of the pilot scan, a decision was made to conduct regular quarterly scans, each covering different trial primary completion date ranges. The inclusion criteria were also adjusted to improve relevance, excluding phase I/II and II trials for non‐rare conditions. As a result, only trials for rare conditions in phases I/II to III, and those for non‐rare conditions in phase II/III and III, were included [15]. The rarity of conditions was checked using the resource, Orphanet [19]. Conducting regular scans led to efforts to optimize the entire scanning process, including the use of existing data, such as condition rarity information available in MInD, and automating checks for inclusion criteria, as well as avoiding duplication by cross‐referencing previously screened records.

The data provided in this paper are collated from multiple scans conducted for clinical trials with primary completion dates from 1 April 2020 to 31 March 2023 (Table 1).

**TABLE 1:** Characteristics for inclusion of clinical trials and their investigative medicines in the repurposed medicines scan.

Criteria	Characteristics for inclusion
Medicinal product	Marketing authorization in the UK/EU
	Off‐patent (generic) repurposed medicines
	On‐patent (branded) repurposed medicines
	Reformulated generic medicinal products
	Biosimilars used outside the marketing authorization of the originator product
	Monotherapy or combination therapy
Clinical trial phase	Phase I/II, II, II/III, and III for rare disease conditions
	Phase II/III and III for non‐rare disease conditions
Trial locations	UK, EU, USA, Australia, or Canada
Trial sponsorship	Non‐commercial sponsors such as academia, hospitals, charities
	Non‐commercial sponsors with industry collaborators
Trial primary completion dates (PCDs)	1 April 2020–31 March 2023 (Dates not applicable to trials sourced from the EU Clinical Trials Register)

## 3 Results

The repurposed medicines scan identified a total of 528 eligible technologies sourced from clinical trial registries. “Technology” refers to a single or combination of medicinal products targeting a specific indication in one or more related trials. For technologies with more than one clinical trial, priority was given to the trial with the highest trial phase and that which had a primary completion date within the specified range (as the “main” trial).

### 3.1 Clinical Trial Characteristics

**FIGURE 1:**
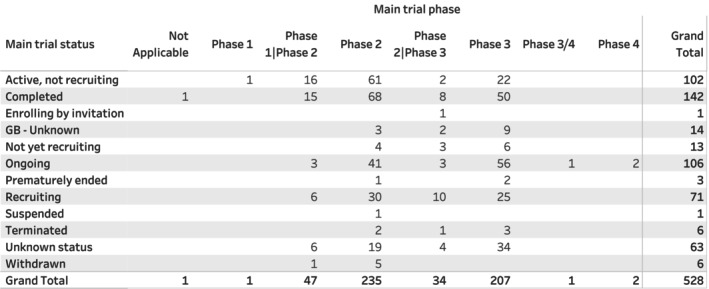
Overview of the clinical trial phases and status of the repurposed technologies.

Analysis of the 528 technologies showed that 93 (17.6%) technologies were no longer in development (terminated, suspended, withdrawn, prematurely ended, or unknown status); 142 (26.9%) technologies had completed, and 293 (55.5%) were in active clinical development (Figure 1).

**FIGURE 2:**
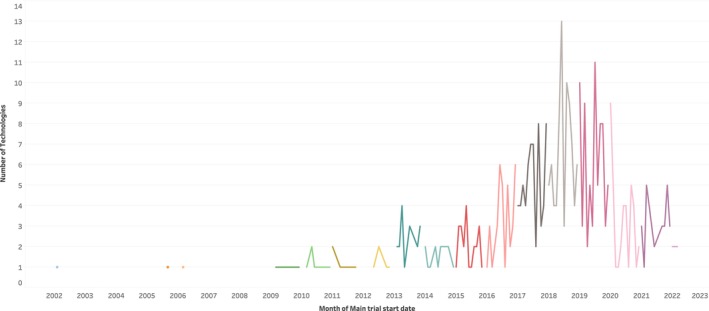
Distribution of trial start dates for the repurposed technologies.

There was a noticeable increase in the number of eligible clinical trials that started between 2017 and late 2020, with a total of 252 trials starting during this period. Earlier than 2010, there were about six eligible trials evaluating repurposed indications according to the inclusion criteria (Figure 2).

**FIGURE 3:**
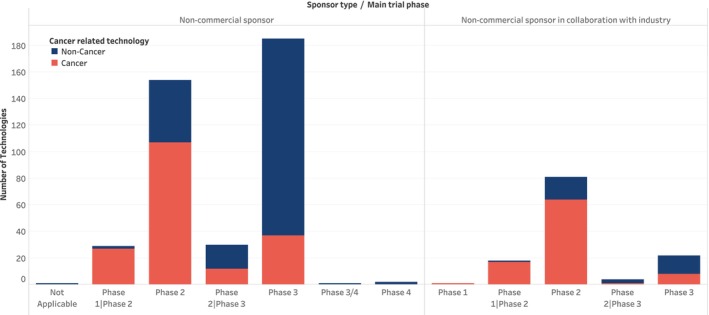
Distribution of trial phases, broad indications, and funder type for the repurposed technologies.

Further classification of the technologies by funder type or sponsorship revealed that 402 (76%) technologies were reported in trial registries as being “solely” sponsored by non‐commercial organizations, while 126 (24%) technologies were reported as sponsored by non‐commercial organizations in collaboration with industry (Figure 3). It is important to note, however, that industry involvement cannot be definitively ruled out in clinical trials reported as “solely” sponsored by non‐commercial organizations. Comparisons between technologies sponsored “exclusively” by non‐commercial organizations and those developed through industry collaborations revealed distinct patterns in their targeted indications. Industry‐collaborated technologies primarily focused on oncology, while non‐commercially sponsored technologies addressed mostly non‐cancer conditions. Further analysis across both sponsorship types indicated that most technologies aimed at non‐cancer treatments were generally in the later stages of clinical trials, whereas those targeting cancer indications were more often in earlier trial phases (Figure 3).

### 3.2 Classification of the Repurposed Technologies

The identified repurposed technologies were categorized using the IO's classification system for innovative medicines, namely, repurposed technologies in combination; off‐patent/generic repurposed technologies; and branded/on‐patent repurposed technologies [20]. There were 206 (39%) technologies evaluating the synergistic effect of combining repurposed medicines; while 322 (61%) tested medicinal products as monotherapy. The combination therapies were either on‐ or off‐patent repurposed medicines, or both. Further categorization of the 322 monotherapy technologies revealed that 182 (56.5%) tested the efficacy of off‐patent/generic medicinal products in a new indication; while 140 (43.5%) monotherapy technologies evaluated medicinal products still covered under UK patent protection (on‐patent/branded).

### 3.3 Categorization According to Therapeutic Areas and Disease Conditions

**FIGURE 4:**
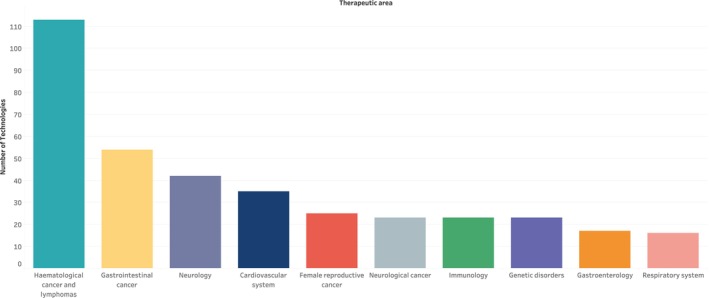
Top 10 therapeutic areas targeted in the repurposed medicines scan.

Further analysis of the 528 technologies showed the range of therapeutic areas explored, with the largest proportions investigating hematological cancers and lymphomas (21.4%), gastrointestinal cancers (10.2%), and neurological disorders (8%) (Figure 4).

**FIGURE 5:**
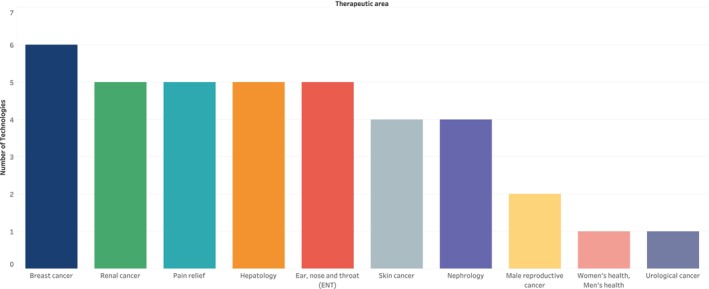
Bottom 10 therapeutic areas targeted in the repurposed medicines scan.

The least investigated therapeutic areas were male reproductive cancers (0.4%), urological cancers (0.2%), and women's and men's health disorders (not otherwise classified) (0.2%) (Figure 5).

**FIGURE 6:**
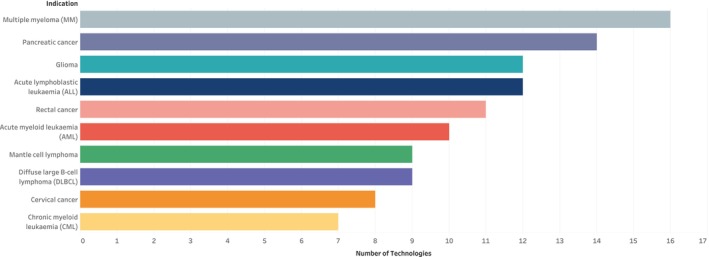
Top 10 indications for rare disease conditions.

Across all the therapeutic areas identified, the technologies were nearly evenly divided between cancer (51.9%) and non‐cancer (48.1%) indications. The data also showed that 357 (67.6%) technologies targeted rare diseases, with multiple myeloma, pancreatic cancer, and glioma being the most treated conditions (Figure 6).

Further classification of the identified technologies by therapeutic class revealed that monoclonal antibodies (159; 30.1%), kinase inhibitors (104; 19.7%), and corticosteroids (41; 7.8%) were the most repurposed classes. Monoclonal antibodies were primarily repurposed for treating cancers and immunological disorders, while kinase inhibitors were most often evaluated for various cancers, particularly hematological cancers and lymphoma. Corticosteroids, on the other hand, were mainly focused on cardiovascular and respiratory system disorders.

## 4 Discussion

The horizon scan for repurposed medicines in development by non‐commercial organizations revealed a total of 528 technologies [medicinal product (or combination of products) for a specified indication] with trial primary completion dates between 1 April 2020 and 31 March 2023. The identified clinical trials revealed the wide range of targeted indications, therapeutic areas, therapeutic classes, and classification of the repurposed technologies.

The remarkable increase in the number of clinical trials that started between 2017 and 2020 highlights the recent attention repurposing is gaining. The medicine repurposing approach was significantly adopted during the COVID‐19 pandemic as a suitable option for the timely discovery of potential treatments for the novel infectious disease [7]. Although clinical trials evaluating treatment options for COVID‐19 were excluded from this scan, the recent increase in number of clinical trials on repurposed medicines shows the high acceptance of the concept to address treatment need.

It must be noted that 402 (76%) technologies were reported as “solely” sponsored by a non‐industry organization such as academia, hospitals, or charities, though industry involvement cannot be definitively ruled out. Lack of industry collaboration may pose legal, financial, or regulatory obstacles to the formal adoption or approval of these medicines in the new indications. As the proposed new indication may be outside the company's business strategy, Fetro recommends an early initiation of an academia‐industry collaboration to establish a “co‐development framework” where expectations and risks are effectively managed and mitigated [21]. However, companies may intend to initiate a line extension for a new indication as part of their business strategy for patent extension; therefore, collaboration with academia may be refused [10, 21].

The IO developed a novel evidence‐based approach to classify innovative technologies, and this was employed to categorize the innovative medicines identified in the repurposed medicines scan [20]. The classification for this scan includes repurposed technologies (combinations) [either on‐ or off‐patent, or both]; off‐patent/generic repurposed technologies; and branded/on‐patent repurposed technologies. Most repurposed technologies in combination treated rare disease conditions (mostly rare cancers) by evaluating the efficacy of the synergistic effect of combination therapies. Some of the targeted rare cancers include acute myeloid leukemia, diffuse large B‐cell lymphoma, and pancreatic cancer. Similarly, most of the repurposed monotherapy technologies treated rare disease conditions, with nearly equal distribution among rare cancers and rare non‐cancers. This is supported by findings by van den Berg et al., which revealed that medicine repurposing for rare diseases is a significant area of research for non‐commercial organizations such as academia and not‐for‐profit stakeholders [22]. There are huge cost implications in the discovery and development of novel therapies for rare diseases, as only 5% of pharmaceutical companies are investing in this area. Repurposing, therefore, provides a faster and economical way to address this high unmet need [9].

Repurposing represents a dynamic medicine development approach for disease areas with high mortality rates and limited treatment options. The scan revealed a diverse range of targeted therapeutic areas, with hematological cancers and lymphomas being the most frequently targeted. Hematological cancers have both high incidence and mortality rates, supporting the impetus for the development of more effective treatment options [23]. The hematological cancers most targeted include acute myeloid leukemia, chronic lymphocytic leukemia, and multiple myeloma. The second most treated therapeutic area was gastrointestinal cancers, in which 54 technologies (10.2%) were evaluated as either monotherapies or combination therapies. Most cases of gastrointestinal cancers are diagnosed at an advanced stage, where the prognosis is poor and survival rate greatly reduced [24]. Chemotherapy, notably combination therapy, has been reported to improve patients' survival and quality of life [24, 25]. This combination of high unmet need and potential for combination therapies to improve outcomes is likely driving the repurposing activity in this space, with the scan revealing that 36 of the 54 technologies (70.6%) treating gastrointestinal cancers were combination therapies. This trend of exploring combination therapies in areas of high unmet need merits further research. As more outcome data are collated, prediction of most promising combinations may become feasible.

A significant number of technologies treated neurological disorders (excluding neurological cancers), which was the third most targeted therapeutic area. The disorders targeted include amyotrophic lateral sclerosis (ALS), multiple sclerosis (MS), and Alzheimer's disease. Treatment for neurological disorders requires a multi‐disciplinary approach to reduce or manage symptoms to improve patients' quality of life and reduce the global health burden [26]. As there is currently no cure for ALS, MS or Alzheimer's disease, researchers continue to explore different therapies to slow disease progression or manage symptoms [27, 28, 29].

The therapeutic or disease areas least investigated in the scan, with six or fewer technologies, include breast cancer; ear, nose, and throat; hepatology; pain relief; renal cancer; nephrology; skin cancer; male reproductive cancer; urological cancer; and women's and men's health. Further work is required by industry and non‐industry into the development of new treatment options for these therapeutic areas. There may have been off‐label use of different medicinal products to treat some of these disease conditions, and these treatments may have been based on the clinicians' discretion; however, there is need for off‐label benefits to be explored in pivotal clinical trials to potentially lead to licensing, and eventually, facilitate more equitable patient access [13].

Monoclonal antibodies (such as pembrolizumab and obinutuzumab), kinase inhibitors (namely, ibrutinib and carfilzomib), and corticosteroids (including dexamethasone and prednisone) were the top three drug classes being repurposed either as monotherapy or in combination with other medicinal products to treat the various therapeutic areas. The monoclonal antibodies targeted mostly cancers and immunological disorders. The diverse modes of action of monoclonal antibodies to directly target tumor cells, while inducing anti‐tumor immune response [30], make them promising candidates for these disease areas. The mechanism of action of kinase inhibitors to block the growth and survival of tumors [31] was explored as repurposed candidates to treat various cancers, most commonly hematological cancers and lymphomas. Corticosteroids (synthetic analogues of natural steroid hormones used for their anti‐inflammatory, immunosuppressive, and vasoconstrictive effects) [32] were evaluated as monotherapies to treat cardiovascular or respiratory system disorders, while their use in various combinations with monoclonal antibodies and/or kinase inhibitors was targeted at oncology indications.

Although every effort was made to ensure accuracy and completeness of the data, the manual, semi‐automated, and automated screening, and classification processes utilized in this study may mean some omissions are likely. Despite obvious limitations such as the specific inclusion criteria, these data show a rich and varied landscape of research in medicine repurposing, across a range of interventions, indications, and trial logistics.

## 5 Conclusion

For a new medicine to be approved for use, regulatory authorities issue a MA after a series of procedures to evaluate its safety and efficacy. For repurposed medicines, the MA holder is required to file a variation, line extension, or submit a new application for the new indication. However, in the case of repurposing by non‐commercial organizations such as academia, support will be needed in the absence of a collaborating company. Additionally, a MA or product license for a medicine does not necessarily mean patient access, hence further support is required to facilitate the adoption of the repurposed medicine into the health service for equitable patient access.

One purpose of the horizon scan for repurposed medicines is to support the identification of potential candidate medicines, which will be assessed for suitability for the NHS England Medicines Repurposing Programme (MRP) [13]. Subsequent scans will provide systematic intelligence on future repurposing opportunities (an early alert process), which will allow the MRP ongoing early intelligence from which to assess and prioritize medicines that can benefit from tailored support toward licensing and adoption into the NHS. This support has the potential to overcome some of the barriers currently faced by non‐commercial organizations. Support may also be provided through identifying a route for fast‐track evidence generation and synthesis where evidence gaps are identified for prioritized medicine, facilitating licensing, or national policy or guidelines [13].

This horizon scanning intelligence also offers audiences an opportunity to look strategically at activity and identify areas for collaboration and further research. Despite challenges which can prevent repurposing research and consequently delay patient access to effective medicines, there is potential for a systematic and transparent horizon scanning system to offer intelligence insights and support strategic research collaborations [12]. The Innovation Observatory has expanded this work and integrated the identification, filtration, and prioritization of repurposed medicines into its routine horizon scanning for innovative health technologies. This intelligence has been provided in an interactive living dashboard [15] on the Innovation Observatory website as a source of intelligence on repurposed medicines, as well as to provide valuable insights into innovation trends, gaps, and areas of unmet medical need [15]. This systematic approach will provide ongoing evidence on future repurposing opportunities of benefit to the NHS and the wider research ecosystem.

## Author Contributions

S.A., R.F., A.I., R.P., and A.O. wrote the manuscript. S.A., R.F., and D.C. conceived the design. R.F., A.I., A.O., and R.P. contributed to data collection. S.A. and R.P. analyzed and interpreted the data. All authors provided critical feedback and helped shape the research, analysis, and manuscript.

## Conflicts of Interest

The authors declare no conflicts of interest.

## Data Availability

The data that support the findings of this study are openly available on the NIHR Innovation Observatory website at [https://io.nihr.ac.uk/news/dashboardpages/repurposed‐medicines‐dashboard/], reference number [15].
